# Incidence and predisposing factors for the development of disturbed glucose metabolism and diabetes mellitus after intensive care admission: the DIAFIC study

**DOI:** 10.1186/cc14446

**Published:** 2015-03-16

**Authors:** S Van Ackerbroeck, K Janssens, P Jorens, T Schepens, W Verbrugghe, V Van Hoof, L Van Gaal, C De Block

**Affiliations:** 1 University Hospital Antwerp, Edegem, Belgium

## Introduction

Stress hyperglycaemia (SH) is commonly observed during hospitalisation in the ICU and adversely influences outcome [1]. When SH occurs in previously nondiabetic patients, this might reflect a latent disturbance of glucose metabolism and predict future risk of diabetes. We wanted to assess the incidence of disturbed glucose metabolism (DGM) and identify predictors for future diabetes risk. This could support timely diagnosis, prevention, and early treatment of impending diabetes mellitus (DM).

## Methods

In this prospective observational study, we enrolled 338 patients without known DM, who were admitted for at least 36 hours to the ICU of the Antwerp University Hospital between September 2011 and March 2013. A 75 g oral glucose tolerance test was performed 6 to 9 months post ICU admission to screen for disturbed glucose metabolism. Furthermore, we examined whether post-discharge glucose disturbances could be predicted by the FINDRISC questionnaire [2], patient demographics, comorbidities, HbA1c at ICU admission, and by parameters related to ICU stay (glucose parameters, insulin need, caloric intake, disease severity).

## Results

In total, 246 patients (73%) experienced SH during their ICU stay. Eight months post ICU admission, glucose metabolism was disturbed in 119 (35%) subjects. Of these, 27 (8%) had impaired fasting glucose, 43 (13%) had impaired glucose tolerance, 25 (7%) had impaired fasting glucose and impaired glucose tolerance, and 24 (7%) were diagnosed with DM. A disturbed glucose metabolism tended to be more prevalent in subjects who experienced SH during ICU stay as compared with those without SH (38% vs. 28%, *P *= 0.065). HbA1c on admission correlated with the degree of SH (*r *= 0.308, *P *< 0.001). The FINDRISC score (9.5 vs. 11, *P *= 0.001), SAPS 3 score (median of 42 in both groups, *P *= 0.003) and daily caloric intake during ICU stay (222 vs. 197, *P *= 0.011) were associated with a DGM.

## Conclusion

Stress hyperglycaemia is frequent in nondiabetic patients and has a tendency towards future disturbances in glucose metabolism and DM. Glucose metabolism was disturbed in 35% of subjects 8 months post ICU admission, of whom 7% was diagnosed with diabetes mellitus. Predictors of elevated risk included a high FINDRISC score, high SAPS 3 score, and a lower daily caloric intake during ICU stay.
